# Diarrhetic Shellfish Toxin Monitoring in Commercial Wild Harvest Bivalve Shellfish in New South Wales, Australia

**DOI:** 10.3390/toxins10110446

**Published:** 2018-10-30

**Authors:** Hazel Farrell, Penelope Ajani, Shauna Murray, Phil Baker, Grant Webster, Steve Brett, Anthony Zammit

**Affiliations:** 1 NSW Food Authority, 6 Avenue of the Americas, Newington, NSW 2127, Australia; phil.baker@dpi.nsw.gov.au (P.B.); grant.webster@dpi.nsw.gov.au (G.W.); anthony.zammit@dpi.nsw.gov.au (A.Z.); 2 Climate Change Cluster (C3), University of Technology Sydney, 15 Broadway, Ultimo, NSW 2007, Australia; penelope.ajani@uts.edu.au (P.A.); shauna.murray@uts.edu.au (S.M.); 3 Microalgal Services, 308 Tucker Rd, Ormond, VIC 3204, Australia; algae@bigpond.com

**Keywords:** diarrhetic shellfish toxins, *Dinophysis*, wild harvest, bivalve shellfish, pipis (*Plebidonax deltoides*), Sydney rock oyster (*Saccostrea glomerata*)

## Abstract

An end-product market survey on biotoxins in commercial wild harvest shellfish (*Plebidonax deltoides*, *Katelysia* spp., *Anadara granosa*, *Notocallista kingii*) during three harvest seasons (2015–2017) from the coast of New South Wales, Australia found 99.38% of samples were within regulatory limits. Diarrhetic shellfish toxins (DSTs) were present in 34.27% of 321 samples but only in pipis (*P. deltoides*), with two samples above the regulatory limit. Comparison of these market survey data to samples (phytoplankton in water and biotoxins in shellfish tissue) collected during the same period at wild harvest beaches demonstrated that, while elevated concentrations of *Dinophysis* were detected, a lag in detecting bloom events on two occasions meant that wild harvest shellfish with DSTs above the regulatory limit entered the marketplace. Concurrently, data (phytoplankton and biotoxin) from Sydney rock oyster (*Saccostrea glomerata*) harvest areas in estuaries adjacent to wild harvest beaches impacted by DSTs frequently showed elevated *Dinophysis* concentrations, but DSTs were not detected in oyster samples. These results highlighted a need for distinct management strategies for different shellfish species, particularly during *Dinophysis* bloom events. DSTs above the regulatory limit in pipis sampled from the marketplace suggested there is merit in looking at options to strengthen the current wild harvest biotoxin management strategies.

## 1. Introduction

Bivalve shellfish are a major global commodity with current market analysis indicating a strong demand for limited available produce [1]. In a demanding market, consumer confidence is essential to support production increases. A major component of a bivalve shellfish safety program is the management and mitigation of the potential risks from biotoxins. Globally, the impact of algal toxins on shellfish aquaculture is variable. In some regions, there has been an apparent increase in the frequency and intensity of toxic events (e.g., recent paralytic shellfish toxins (PST) events in Tasmania [2,3]) but with effective monitoring and management, the risk of illness outbreaks can be minimised [4,5]. All biotoxin groups are of concern to shellfish safety managers, and more than one toxin group can occur concurrently. In the case of *Dinophysis* spp., certain species can produce diarrhetic shellfish toxins (Diarrhetic shellfish toxins (DSTs): okadaic acid (OA) and dinophysistoxins (DTX)) at very low cell densities (200 cells/L) [6,7,8]. OA, DTX 1, and DTX-3 are diarrheagenic and some OA/DTX analogues have been associated with tumor formation in laboratory studies on rodents [9,10,11]. Diarrhetic shellfish poisoning (DSP) was first described in the late 1970s following human illness outbreaks in Japan [12], yet early reports of gastrointestinal illness suspected as DSP date back to 1961 [7]. The acute symptoms of DSP are generally alleviated within a few days and no fatalities from acute cases of DSP have been recorded. Certain *Dinophysis* species can also produce pectenotoxins (PTX, previously part of the DST complex), although there is no known evidence that PTXs are toxic to humans [5]. Very high concentrations of *Dinophysis* can occur in thin layers and other micro and mesoscale oceanographic structures, which means that species of this genus can be difficult to detect [8,13,14,15]. These difficulties are compounded by the fact that *Dinophysis* species are generally mixotrophic, and the laboratory culture of species of this genus has only recently been achieved [16,17,18,19,20,21,22,23]. Until this development, verifying toxins produced by individual species and understanding the factors affecting toxin production have been challenging.

**Figure 1:**
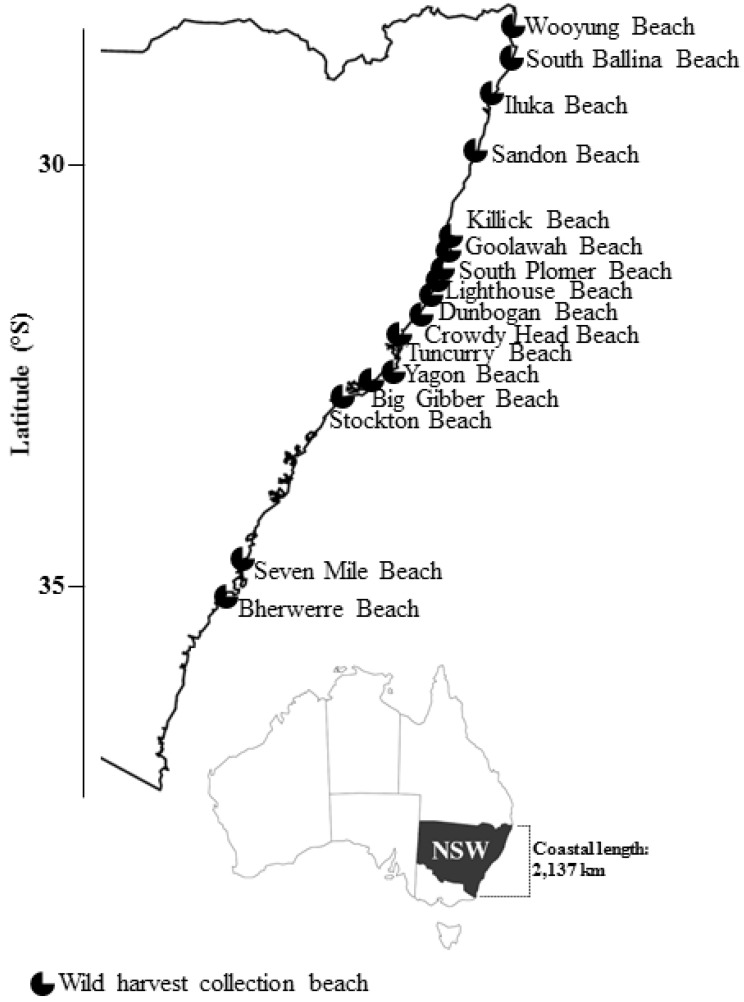
Location of wild harvest beaches in New South Wales (NSW), Australia from which pipis were harvested during the 2015–2017 harvest seasons.

In New South Wales (NSW), Australia, bivalve shellfish aquaculture stretches along >2000 km of coastline with a farm gate value of more than $AUD 47 million per year [24]. The main cultivated species is the native Sydney rock oyster (*Saccostrea glomerata*). Other cultivated species include Pacific oyster (*Magallana gigas* formerly *Crassostrea gigas*), native oyster (*Ostrea angasi*), and blue mussel (*Mytilus edulis*). Seasonal (June–December) wild harvest shellfish collection from open beaches is focused on pipis (‘clams’, *Plebidonax deltoides*) at up to 16 beaches (Figure 1). Gathering of cockles (*Katelysia* spp., *Anadara granosa*) occurs within six oyster harvest areas, and a single operator collects clams (*Notocallista kingii*) through offshore dredging along the NSW south coast (~36°54.5′ S). Under the NSW Marine Biotoxin Management Plan [25], shellfish collected or grown for human consumption in NSW are subject to monitoring (phytoplankton in water adjacent to harvest areas and biotoxins in shellfish flesh) to ensure that the product is safe to eat.

Three types of biotoxins are currently known to occur in NSW (amnesic shellfish toxin (AST), diarrhetic shellfish toxins (DSTs) and paralytic shellfish toxins (PSTs)). In NSW, these toxin groups are routinely monitored (biotoxin testing of shellfish flesh and microscopic analysis of water samples for causative phytoplankton) in locations where shellfish are cultivated and harvested (or collected in terms of wild shellfish) for human consumption. Neurotoxic shellfish toxins (NSTs) and azaspiracid shellfish toxins (AZTs) have not been detected in NSW, or Australia, to date [3,26,27,28,29]. The permissible level of biotoxins in shellfish is regulated in Standard 1.4.1 clause 3 of the Australia New Zealand Food Standards Code [30] (The Code). The limits specified within The Code are similar to the European Union (EU) and the United States of America (USA) regulatory standards (Table 1).

Since the establishment of the current phytoplankton and biotoxin monitoring program by the NSW Food Authority in 2005, all three of the major toxin groups (AST, DSTs, PSTs) have been detected in shellfish tissue in NSW [29]. Biotoxin data from wild harvest beaches have shown detections of DSTs in pipis related to *Dinophysis* spp., with occasional reports of AST (NSW Food Authority 2018, unpublished data). While the NSW dataset did not report the presence of PSTs in pipis, PSTs, DSTs, and AST have been reported in shellfish species (cockles and clams) from similar intertidal or sandy-bottomed marine habitats (e.g., AST: razor clam (*Siliqua patula*) Washington State, USA [33,34,35], DSTs: littleneck clam (*Leukoma staminea*), varnish clam (*Nuttallia obscurata*), manila clam (*Ruditapes philippinarum* syn. *Venerupis philippinarum*) Washington State, USA [34] and PSTs: surf clam (tuatua, *Paphies subtriangulata*) Bay of Plenty, New Zealand [36]).

Historically, in NSW, most phytoplankton toxin-related illnesses have been linked to ciguatoxin in migratory and imported reef fish [37,38,39] rather than bivalve shellfish. To date, no illnesses linked to biotoxins from NSW oyster or mussel aquaculture areas have been reported (NSW Food Authority 2018, unpublished data). Before the establishment of routine monitoring on NSW wild harvest beaches, two illness outbreaks occurred following consumption of pipis. Both outbreaks were associated with DSTs in 1997 (north NSW coast; 102 cases including, 46 anecdotal) [40] and 1998 (mid-north NSW coast; >20 cases) [27,41]. In Australian waters, 36 species of *Dinophysis* have been documented [28,42,43], of which *Dinophysis acuminata* (Claparède and Lachmann), *Dinophysis acuta* (Ehrenberg), *Dinophysis caudata* (Saville-Kent), *Dinophysis fortii* (Pavillard), and *Dinophysis tripos* (Gourret) are known toxin producers, along with *Phalacroma mitra* (syn. *Dinophysis mitra*). Reports of *Dinophysis* and DST events elsewhere in Australia have been few, although the availability of long-term phytoplankton and biotoxin datasets across all Australian states is limited. A single case of DSP from pipis collected from a beach on North Stradbroke Island, Queensland was reported in 2000 [44]. DSTs above the regulatory limit have been reported in pipis from NSW (suspected *D. acuminata*) [45], in oysters from South Australia (*D. acuminata*) [46], and in mussels from Tasmania (*D. acuminata* and *D. fortii*) [47].

Given the frequent reports of DSTs both above and below the regulatory limit in wild harvest shellfish when compared to aquaculture shellfish in NSW [29,45], coupled with an increasing demand and value of pipis [48], further investigation into potential consumer risk from biotoxins was required. In the current study, an end-product survey was carried out over three wild harvest seasons (2015–2017) to evaluate the biotoxin management of wild shellfish harvest operations.

## 2. Results

### 2.1. Wild Harvest Shellfish End-Product Market Survey

Of the samples tested, 99.38% complied with regulatory limits. DSTs were detected only in pipi samples (40.59%, 110 of 271 samples). AST was detected in three pipi and two strawberry clam samples (maximum reported level = 3.50 mg/kg domoic acid (DA)). PSTs were not detected during the survey (Table 2). During the sampling period, two market survey samples exceeded the DST regulatory limit (0.20 mg/kg OA, Lighthouse Beach, Date of harvest (DOH) 19 December 2016, 0.23 mg/kg OA; Stockton Beach DOH 27 September 2017, 0.21 mg/kg OA).

Over the three wild harvest seasons, positive DST results in pipi samples were 82.35 (2015), 22.00 (2016), and 38.33 (2017) % across the state (Table 3). Okadaic acid was the single DST analogue identified during the survey. Examination of the spatial and temporal distribution of positive DST results (Table 4) indicated that positive detections at Stockton Beach (Figure 1) during the 2016 and 2017 wild harvest seasons occurred during weeks 33–39 (Table 4) earlier than beaches further north (weeks 45–52) (Table 4). This spatial pattern was not apparent during 2015 due to a shorter sampling window between November to December (Table 4). Data from 2015 to 2017 demonstrated that positive biotoxin results persisted throughout the wild harvest season once detected at most beaches (Table 4).

### 2.2. Wild Shellfish Harvest Beaches Phytoplankton and Biotoxin Samples

**Figure 2:**
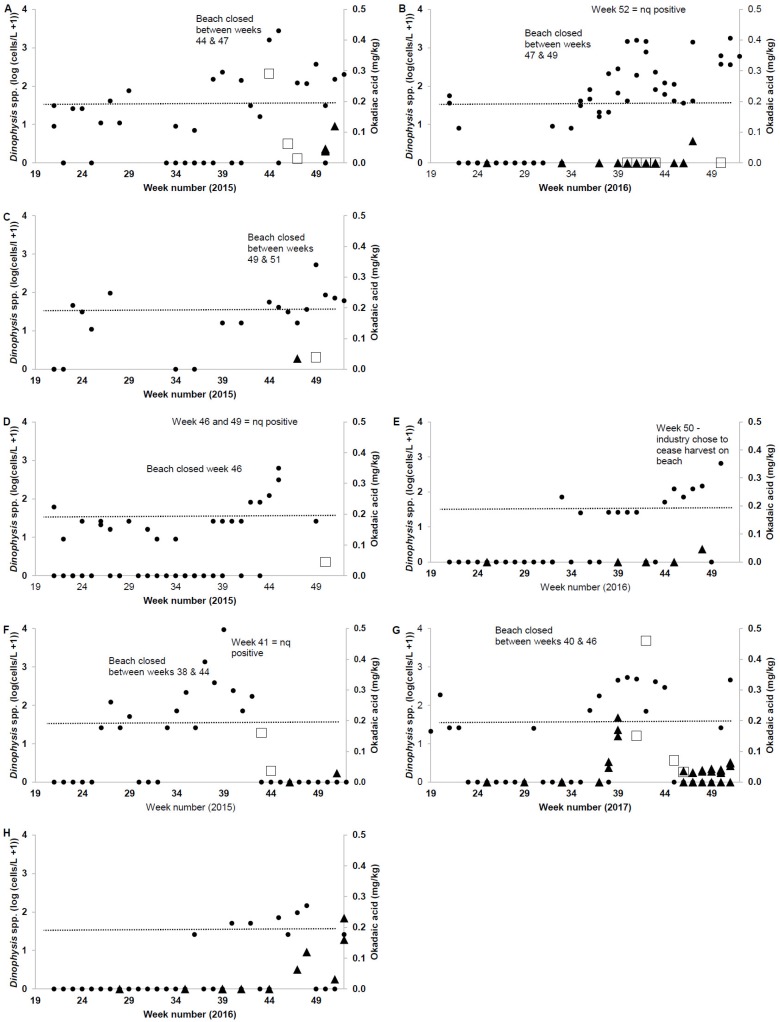
Temporal distribution of *Dinophysis* spp. (log (cells/L + 1), black circle) and okadaic acid (mg/kg) from market survey (black triangle) and routine monitoring (white square) biotoxin samples at South Ballina Beach 2015 (**A**) and 2016 (**B**); Iluka Beach 2015 (**C**); Killick Beach 2015 (**D**) and 2016 (**E**); Stockton Beach 2015 (**F**) and 2017 (**G**); and Lighthouse Beach 2016 (**H**). Note: a zero result is equivalent to <0.025 mg/kg OA, dashed line indicates regulatory limit of 0.2 mg/kg OA, nq = not quantified Wild harvest beaches are listed north to south and their locations are provided in Figure 1.

During the 2015–2017 wild harvest seasons, 1097 phytoplankton samples were collected from sixteen wild harvest beaches (Table 5, Figure 1). The maximum concentration of *Dinophysis* spp. reported was 9330 cells/L from Stockton Beach (Table 5). Seventeen samples from six beaches contained concentrations of *Dinophysis* spp. above the phytoplankton action level (PAL) of 500 cells/L [25] (Table 5). Following the PAL exceedance and the subsequent biotoxin tests conducted, two shellfish (pipi) samples (2015 and 2017) exceeded the regulatory limit for DSTs (South Ballina 0.29 mg/kg OA, 0.03 mg/kg PTX2, October 2015 and Stockton Beach 0.46 mg/kg OA, October 2017) (Table 5, Figure 1). A pattern of elevated *Dinophysis* spp. concentrations detected at Stockton Beach (Figure 1) earlier than beaches further north was apparent (for example refer to Figure 2).

### 2.3. Comparison of Market Survey and Wild Shellfish Harvest Data

Market survey data were available for comparison to pipi wild harvest monitoring data for four (South Ballina Beach, Iluka Beach, Killick Beach and Stockton Beach) of the six locations where the PAL for *Dinophysis* spp. was exceeded (Table 5). For five of the six *Dinophysis* bloom events, biotoxin samples supported the existing biotoxin management plan, and all market survey results were below regulatory limits for DSTs (Figure 2A–F,H). On one occasion (Figure 2G), a biotoxin sample from pipis collected from Stockton Beach on 27 September 2017 (week 39) returned a positive result of 0.21 mg/kg OA. *Dinophysis* spp. concentrations were 448 cells/L in a seawater sample collected Stockton Beach during week 39 (24 September). Cell concentrations above the PAL (500 cells/L) for this group were not reported until week 40 (2 October). Biotoxin samples collected from the beach during weeks 41 (8 October) and 42 (15 October) returned positive results of 0.15 and 0.46 mg/kg OA, respectively (regulatory limit 0.2 mg/kg OA), while cell concentrations appeared to decline (480 and 69 cells/L for weeks 41 and 42, respectively).

At South Plomer Beach (2015) and Bherwerre Beach (2017), in lieu of biotoxin testing, the wild harvest industry chose to cease harvest when the PAL exceedances for *Dinophysis* spp. were reported (Table 5). No market survey samples were collected for either of these beaches.

An alternative comparison was the positive, above regulatory limit, biotoxin result of 0.23 mg/kg OA to phytoplankton results. The positive sample was from Lighthouse Beach (Figure 1) and pipis collected on 19 December 2016. The preceding phytoplankton samples from this beach did not indicate an increase in *Dinophysis* spp. (Figure 2H), with cell concentrations <150 cells/L.

### 2.4. Phytoplankton and Biotoxin Samples from Oyster and Mussel Harvest Areas

Up to ten species of *Dinophysis* were observed in water samples from shellfish aquaculture areas across the state (*D. acuminata*, *D. caudata*, *D. rotundata* (= *Phalacroma rotundatum*), *D. acuta*, *D. tripos*, *D. fortii*, *D. truncata*, *D. schroederi*, *D. mitra* (= *Phalacroma mitra*) and *D. hastata*) (refer Figure S1). *D. acuminata* was the most common species observed. *D. acuminata* was observed in all estuaries except for the Tweed River (Table 6). From the available data, *D. acuminata* was observed to be present in elevated (up to 3200 cells/L) concentrations on the north NSW coast between August and December (Table 6). The second most common species observed was *D. caudata* (Table 6). Maximum concentrations (up to 1500 cells/L) were reported between November and June, but the distribution of *D. caudata* between estuaries was more variable (Table 6). The other *Dinophysis* spp. observed did not exceed the 500 cells/L PAL and these species were generally observed south of 31°38′ S (data not shown). Biotoxin testing from shellfish aquaculture areas during this period did not detect DTX or OA positive results [29] (NSW Food Authority, unpublished data). Pectenotoxin-2 was detected at low concentrations (max 0.036 mg/kg) in two samples from Wonboyn Lake (37°17′ S) [29].

## 3. Discussion

Most wild shellfish harvest in NSW is focused on mid-north and north coast beaches and coincides with seasonal *Dinophysis* events during the Austral spring and summer months. Our study conducted over three consecutive wild harvest seasons in NSW highlighted DSTs as the main concern due to their presence in over one-third of the shellfish samples tested. On two occasions, DSTs were detected above the regulatory limit in the marketplace and suggested that the current wild harvest biotoxin management processes could be strengthened. In the first scenario, cell concentrations at Lighthouse Beach did not exceed the PAL for *Dinophysis* spp. This elevated DST result was reported following the annual closure of the harvest season, and further phytoplankton or biotoxin samples were not available to evaluate how or if the bloom progressed. During the second incident, *Dinophysis* concentrations at Stockton Beach did not exceed the PAL until a week after a DST result above the regulatory limit was detected. Both circumstances resulted in shellfish above the regulatory limit for DSTs entering the market. While no illnesses were reported related to these events, this study was an opportunity to consider improvements in the current wild harvest biotoxin management plans. While this study highlights the potential risk of DST contaminated product entering the marketplace, the emphasis on other biotoxin groups could be redirected if there was a shift in dominant harmful phytoplankton near existing beaches, or if the industry chose to relocate to a location where different biotoxins were present.

*Dinophysis* spp. cell densities reported from wild harvest beaches varied along the NSW coast. Without a full understanding of how pipis uptake and depurate DSTs it is difficult to elucidate the patterns involved. The dynamics of intertidal habitats are not readily comparable to studies of uptake and depuration of DSTs in mussels and oysters in planktonic environments (e.g., Pitcher et al. [49], Wallace 2011 [47]). As depuration of biotoxins from clams and pipis tends to be slower than oysters and mussels [5,36,50], this may result in prolonged periods where positive toxins are detected. As in other surf clams, pipis feed via a siphon. In the butter clam (*Saxidomus giganteus*) PSTs have been found to accumulate and be retained in the siphon [51], and we hypothesise that a similar mechanism could be occurring in pipis. Moreover, the uptake and depuration of toxins varies substantially between bivalve species. The northern quahog (*Mercenaria mercenaria*) can selectively feed during exposure to *Alexandrium* by retracting its siphon and closing its valve [52,53]. In contrast, selective feeding of *Dinophysis* spp. has been observed via examination of the gut of the Mediterranean mussel (*Mytilus galloprovincialis*) [54]. There is no information published on the uptake or depuration dynamics of DSTs by pipis specifically, and more investigation is required.

Other possible reasons for the disparities between the beach monitoring data and market survey data in this study could be attributed to the natural non-homogenous distribution of phytoplankton, toxin variability between individual cells or strains of *Dinophysis* spp., the current phytoplankton net sampling technique or a combination of these. In addition, knowledge of the bloom dynamics involved are limited by phytoplankton data reported to genus level only and lack of simultaneous environmental data (e.g., temperature, salinity, turbidity, current data). The current study demonstrated that weekly phytoplankton sampling alone was not sufficient to ensure that shellfish product with DSTs above the regulatory limit were not harvested. At Stockton Beach, the beach was closed to harvest following the report of the above DST regulatory limit market survey result. DST concentrations, both below and greater than twice the regulatory limit, were reported from shellfish (pipi) samples collected at Stockton Beach in the following weeks. The incorporation of routine biotoxin monitoring into the wild harvest monitoring program would improve understanding of variability in toxin concentrations over short time periods and unknown differences between toxic strains of *Dinophysis* in this region. Furthermore, and pending an appropriate risk analysis, a shift to a seasonal quota system for the NSW wild harvest shellfish industry could allow fishers to collect pipis during lower risk periods.

Concurrent phytoplankton data from shellfish aquaculture areas demonstrated that *D. acuminata* was the predominant *Dinophysis* species occurring in NSW estuaries, with greatest concentrations observed in estuaries north of 32°42′ S during the Austral spring and summer. Ajani et al. [55] have also reported peaks of *Dinophysis* cell concentrations during summer (January) offshore of Sydney (Port Hacking). This information is comparable with other field studies of *Dinophysis* in Australia. Takahashi et al. [56] found that on North Stradbroke Island, Queensland, Australia that *Dinophysis* spp. were more common during warmer months, with *D. acuminata* only reported on open beaches between November and January. Reports on *Dinophysis* spp. in Australian waters have shown the genus to be “common but rarely abundant” [3,26,27,28,57]. A study on *Dinophysis* spp. within the upper reaches of the Hawkesbury river estuary demonstrated a similar seasonality to this study with *D. acuminata* and *D. caudata* having greatest abundances in spring and summer/autumn, respectively [57]. While phytoplankton sampling was by undertaken different methods in estuaries and beaches, data from the present study supports the view that *D. acuminata* was the main source of DSTs in pipis. More data are required to substantiate this extrapolation, but it is a likely explanation given that elevated concentrations of *Dinophysis* spp. and the presence of OA in wild harvest samples occurring within a similar season (early October onwards). Historical illnesses linked to DSTs in pipis from NSW were assumed to be caused by *D. caudata* and pectenotoxin-2 and pectenotoxin-2-seco acids [44]. It was later clarified that OA derivatives from *D. acuminata* had been the causative agents [58]. Additionally, *Prorocentrum* spp. were not considered to be a cause of DSTs in NSW [29]. While linked to toxin production historically elsewhere, *Prorocentrum* spp. have not been found to be toxin producing in NSW to date [29]. Negative DST results in estuarine shellfish harvest areas suggest that *Dinophysis acuminata* blooms in NSW originate offshore or along the coastline. Ajani et al. [26] also showed that *D. acuminata* was significantly more abundant at downstream sites when compared to upstream sites within NSW estuaries, thus, supporting the oceanic origin hypothesis. While further investigation is needed into if and how *Dinophysis* blooms are transported into NSW estuaries, similar along-shore transport has been observed for *Dinophysis* in other locations (e.g., Spain/Portugal [59], Ireland [14]).

More than twelve years of routine phytoplankton and biotoxin data from estuaries has demonstrated a low risk of DSTs and other phytoplankton toxin groups for NSW oyster consumers, and the current monitoring in estuaries is effective at minimising consumer risk [29]. Mussels generally accumulate DSTs more readily than oysters (e.g., Pitcher et al. [49]) and while during this study pipis were the main species affected by DSTs in NSW, oyster samples from South Australia have shown DSTs above the regulatory limit [46]. Worldwide, new cases and outbreaks of DST are still occurring (e.g., British Columbia [60], China [61], Brazil [62]). While the occurrence of DSTs has been variable in NSW [3,29,45], a DST event in Tasmania during 2016 was responsible for a recall of mussels from a location that was not previously known to be impacted by DSTs [63]. In a changing environment, where phytoplankton blooms are seemingly more frequent and intense [7,8,64,65], management strategies need to be adaptable to manage the potential risks for shellfish consumers. The use of sentinel species or passive samplers may be an option for risk management, but these techniques can have limitations depending on the harvest area conditions or targeted toxins (e.g., [66,67]). Historically, phytoplankton and biotoxin monitoring programs have been established following illness outbreaks (e.g., monitoring of wild harvest beaches in NSW following DSP events and Thermaikos Gulf in Thessaloniki, Greece [68]) but long-term data can help inform existing shellfish safety programs. For example, at the Coorong harvesting area in SA, routine biotoxin testing during the pipi harvest season occurs monthly at one location. This regime increases to a fortnightly sampling frequency for biotoxins at three sample locations during upwelling events, which can impact phytoplankton production (C. Wilkinson, *pers comm*).

In NSW, due to extended consecutive DST positive results during *Dinophysis* bloom events, pipi harvesters tend to relocate and operate in other open status beaches rather than continue testing at ‘positive’ beaches. Protection of consumers from biotoxin-related illnesses is critical in maintaining customer confidence in shellfish produce and to safeguard the growing wild harvest shellfish industry in NSW. More data are required to understand *Dinophysis* bloom dynamics and to substantiate that *D. acuminata* is the main source of DSTs in pipis in NSW. The notable occurrences of DST positives presented in this study suggest that there is merit in augmenting the current testing regime on wild harvest beaches by adopting a regime that includes frequent biotoxin monitoring. The development of more cost-effective, rapid and reliable test methods would improve risk management while maximising harvesting opportunities for industry.

## 4. Materials and Methods

### 4.1. End-Product Market Survey

#### 4.1.1. End-Product Sample Collection (Shellfish)

End-product market survey shellfish sample collection focused on Sydney Fish Market, Sydney, Australia, as most wild harvest shellfish collected in NSW is consigned through the market for auction. Between November 2015 and December 2017, 323 wild harvest shellfish samples were collected (Table S1). Wild shellfish harvest is focused on mid-north and north coast beaches in NSW (Figure 1, Figure S2). Pipis were the predominant wild harvest shellfish available for sale at the time of sampling (Table 2). Sampling frequency was increased from monthly to weekly between September and December during each year (Table S1) in line with historical phytoplankton data and positive DST detections from wild harvest beaches in NSW [26,45]. Depending on the amount of wild harvest stock on sale, more than one sample from a wild harvest beach was collected, as often multiple licensed individuals collect shellfish on the same beach. In addition, on some beaches there were more than one wild harvest collection group operating (Table S2). Each shellfish sample was a homogenate of the soft tissue of 15–20 individual shellfish (min. 100 g of meat was collected). The samples were kept chilled and either delivered to a National Association of Testing Authorities (NATA) accredited biotoxin laboratory (Symbio Laboratories, Sydney) within 1 h of collection or frozen (−20 °C) for later analyses.

#### 4.1.2. Biotoxin Testing of Shellfish Samples

All end-product market survey samples were screened for PSTs by high performance liquid chromatography (HPLC) [69]. Initial screening for PSTs included the analogues STX, GTX2,3, C1,2, GTX5, NEO, dcNEO, and GTX1,4. If a positive result was reported, pre-column oxidation was used to confirm concentrations of STX, GTX2,3, C1,2, GTX5, dcSTX, dcGTX2,3, NEO, dcNEO, GTX1,4, C3,4. AST (domoic acid (DA)), and DSTs (OA, dinophysistoxin 1 (DTX-1), dinophysistoxin 2 (DTX-2)), and pectenotoxin 2 (PTX-2) by liquid chromatography tandem mass spectrometry (LCMS/MS) [70,71,72]. The lipophilic toxins cylindrospermopsin, gymnodimine, spirolide 1, azaspiracid 1, azaspiracid 2, azaspiracid 3, and yessotoxin were also included as part of the LCMS/MS screen [71]. Positive toxin results were equivalent to ≥1.00 mg/kg DA (AST), ≥0.25 mg/kg OA equivalents (DSTs) and ≥0.10 mg/kg STX equivalents (PSTs).

### 4.2. Routine Monitoring at Shellfish Harvest or Collection Areas

#### 4.2.1. Sample Collection for Phytoplankton Analyses (Water)

Phytoplankton and biotoxin data collected within the same timeframe as the market survey samples (November 2015–December 2017) from both wild harvest beaches and shellfish aquaculture areas were compared to the market survey data. The current NSW Food Authority monitoring program for phytoplankton and biotoxins in NSW distinguishes between aquaculture and wild shellfish harvest areas. The location of phytoplankton and biotoxin sample sites are designated as representative of the water filtered by shellfish in each location [25,73].

**Figure 3:**
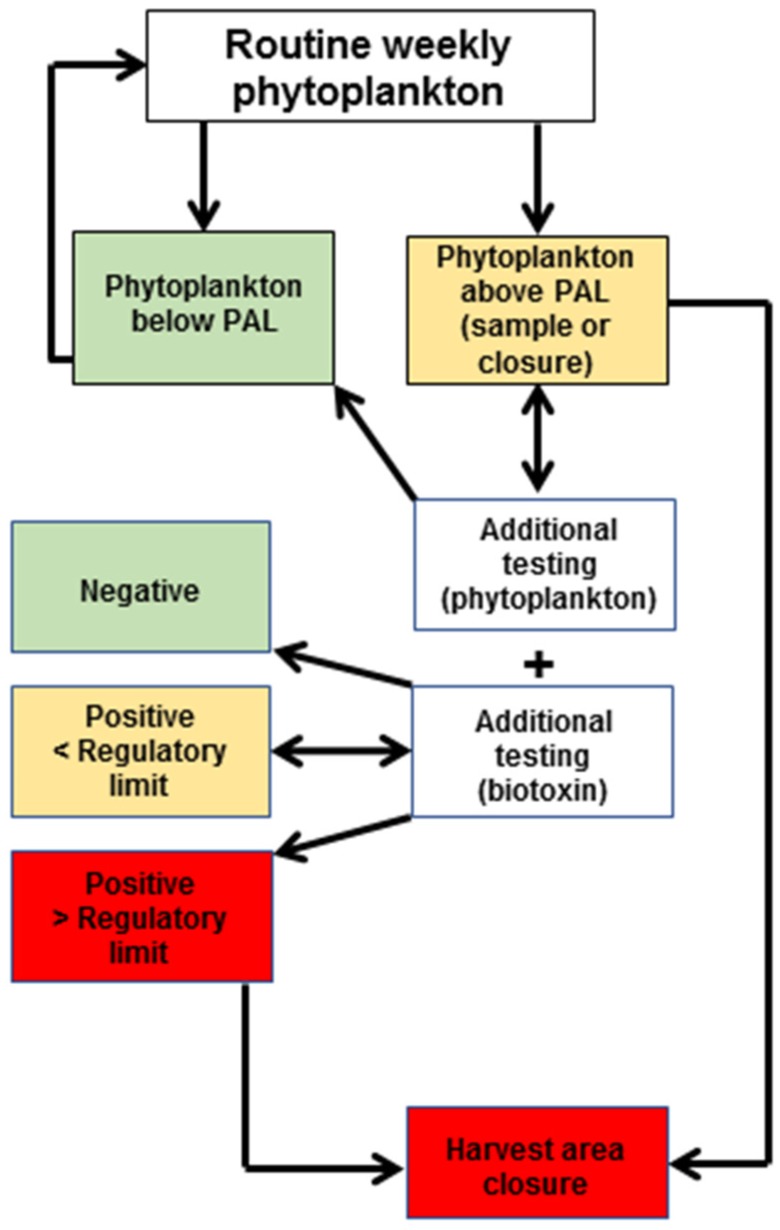
Phytoplankton and biotoxin monitoring program for wild harvest beaches in NSW [25].

Routine phytoplankton samples for wild harvest shellfish and shellfish aquaculture areas are collected weekly and fortnightly during the open harvest status, respectively. Samples are collected by trained shellfish industry members. During each wild harvest season, a weekly phytoplankton sampling program was followed. Seawater samples (~50 L = 5 × 10 L buckets of seawater) were concentrated by a 20 µm mesh phytoplankton net (to ~500 mL) and preserved with Lugol’s Iodine. When open for harvest, shellfish aquaculture areas were subject to the collection of fortnightly discrete sub surface (0.5 m) estuarine water samples (500–1000 mL), with a phytoplankton net surface drag sample collected at each sample site. Both samples were preserved with Lugol’s Iodine for later analysis by microscope for potentially harmful species listed in Appendix 9 of the NSW Marine Biotoxin Management Plan [25]. Sub-samples (1 mL) of concentrated seawater samples from wild harvest beaches were analysed. Note that phytoplankton concentrations reported from wild harvest beach samples were identified to genus level only. Estuarine water samples from shellfish aquaculture areas were concentrated by gravity-assisted membrane filtration (5 µm) prior to analysis. Simultaneous phytoplankton net haul samples were utilised to assist with identification. As a cost saving measure, if a PAL is reported, industry may choose to delay sampling. For example, the wild harvest beaches can be closed for collection until subsequent phytoplankton and biotoxin testing demonstrates that any contamination has ceased (Figure 3).

#### 4.2.2. Sample Collection for Biotoxin Testing (Shellfish)

On wild harvest beaches, biotoxin samples were collected when a PAL was exceeded (for example >500 cells/L *Dinophysis* spp.) [25]. Biotoxin sampling occurred weekly if a wild harvest beach was open and toxin levels were below regulatory limits. Shellfish aquaculture areas were also subject to routine monthly biotoxin sampling when the harvest areas were open for harvest. Shellfish tissue samples (12–14 individuals, min. 100 g flesh) were collected, shucked and frozen prior to dispatch for biotoxin analysis at a NATA accredited laboratory. Shellfish samples from aquaculture or wild harvest areas were analysed for biotoxins either by Jellett screening (Jellett Rapid Testing Ltd., Nova Scotia, Canada) or by the qualitative methods, as above.

## Figures and Tables

**Table 1:** Regulatory limits for biotoxins in bivalve molluscs from the European Union (EU), United States of America (USA) and Australian legislation.

Toxin Group	EU [31]	USA [32]	Australia [30]
Amnesic shellfish toxin (AST, domoic acid equivalent)	20 mg/kg	20 mg/kg	20 mg/kg
Diarrhetic shellfish toxins (DSTs, okadaic acid equivalent)	0.16 mg/kg	0.16 mg/kg	0.20 mg/kg
Paralytic shellfish toxins (PSTs, saxitoxin equivalent)	0.80 mg/kg	0.80 mg/kg	0.80 mg/kg
Neurotoxic shellfish toxins (NSTs, brevetoxin-2 equivalent)	n/a ^1^	0.8 ppm or 200 MU/kg	200 MU/kg
Azaspiracid shellfish toxins (AZTs)	0.16 mg/kg	0.16 mg/kg	n/a ^2^

^1^ not applicable, there are currently no EU regulatory limits for NSTs; ^2^ not applicable, AZTs have not been detected in Australia. If identified, international regulatory limits would apply.

**Table 2:** All NSW wild harvest shellfish samples collected as part of the end-product market survey November 2015–December 2017. Each sample was a homogenate of the soft tissue of 15–20 individual shellfish.

Shellfish Type	*n* = 321	AST (No. Positive/Above Regulatory Limit)	DST (No. Positive/Above Regulatory Limit)	PST (No. Positive/Above Regulatory Limit)
Pipis (*Plebidonax deltoides*)	271	3/0	110/2	0/0
Cockles (*Katelysia* spp. *Anadara granosa*) ^1^	47	0/0	0/0	0/0
Strawberry clam (cockle) (*Notocallista kingii*)	3	2/0	0/0	0/0

^1^ Gymnodimine was detected in four samples (0.028, 0.041, 0.041, 0.072 mg/kg).

**Table 3:** All pipi samples (positive DST detections and total number of samples) from wild harvest beaches collected as part of the end-product market survey during the 2015, 2016, and 2017 wild harvest seasons. Each sample was a homogenate of the soft tissue of 15–20 individual shellfish.

Wild Harvest Beach (North–South)	2015 (Positive/Total)	2016 (Positive/Total)	2017 (Positive/Total)
South Ballina Beach	7/7	1/16	0/15
Iluka Beach	1/1	-	0/10
Killick Beach	-	1/5	3/12
Goolawah Beach	12/12	2/7	6/14
South Plomer Beach	-	0/1	-
Lighthouse Beach	13/13	5/11	2/3
Dunbogan Beach	-	3/6	6/7
Crowdy Head Beach	6/9	3/11	4/12
Tuncurry Beach	-	0/1	-
Yagon Beach	2/5	1/19	5/12
Big Gibber Beach	-	0/7	-
Stockton Beach	1/2	6/16	20/35
Unconfirmed ^1^	0/2	-	-
Total	42/51	22/100	46/120

^1^ Supplying co-op notified regarding labelling requirements.

**Table 4:**
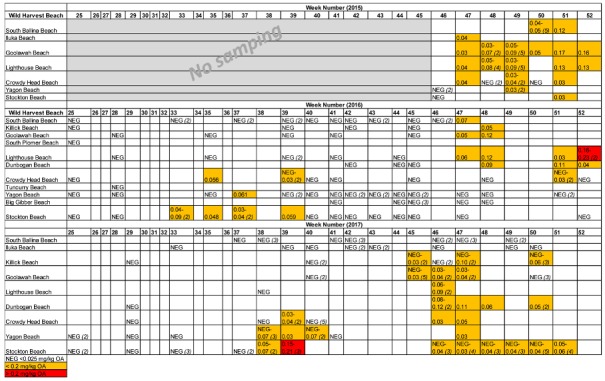
Spatial and temporal distribution of okadaic acid (mg/kg OA) in pipi samples collected as part of an end-product wild harvest market survey (2015, 2016 and 2017 harvest seasons). The locations and week numbers listed correspond to the beach where the shellfish were collected and the harvest date, respectively. Where more than one sample was collected the range of results are provided, with the number of samples noted in brackets. For clarity of presentation, the okadaic results are round to two decimal places. The locations of wild harvest beaches listed each year in the order of north to south are shown in Figure 1.

**Table 5:** Summary of monitoring data (phytoplankton and biotoxin) from wild harvest beaches collected during the 2015, 2016, and 2017 wild harvest seasons.

Wild Harvest Monitoring Data	2015	2016	2017	All
No. of phytoplankton samples collected	310	411	376	1097
No. of phytoplankton samples >500 cells/L *Dinophysis* spp.	7	8	2	17
South Ballina Beach/Max cells/L *Dinophysis* spp.	2760	1760	-	-
Iluka Beach/Max cells/L *Dinophysis* spp.	520	-	-	-
Killick Beach/Max cells/L *Dinophysis* spp.	625	650	-	-
South Plomer/Max cells/L *Dinophysis* spp.	825	-	-	-
Stockton Beach/Max cells/L *Dinophysis* spp.	9330	-	530	-
Bherwerre Beach/Max cells/L *Dinophysis* spp.	-	-	500	-
No. of DST tests	10	10	6	26
No. of DST positive results	8 ^1^	1 ^2^	4	4
No. of DST results > 0.2 mg/kg OA	1	0	1	2

^1^ Three positive test results were not quantified; ^2^ One positive test result was not quantified.

**Table 6:** Summary of *D. acuminata* and *D. caudata* reported from shellfish (oyster and mussel) producing estuaries north to south along the NSW coastline (November 2015–December 2017). Observations of cell concentrations above 500 cells/L are highlighted in bold, corresponding to the PAL for *Dinophysis* spp. applied to routine monitoring for wild shellfish harvest beaches.

Estuary (North–South)	Latitude (S)	No. of Sample Sites	Total Samples Per Site (November 2015–December 2017)	*D. acuminate* (No. of Observations/Max. Cells/L /Month of Max. Concentration)			*D. caudate* (No. of Observations/Max. Cells/L /Month of Max. Concentration)		
Tweed River	28°10′	1	45	-	-	-	3	**700**	December
Richmond River	28°53′	1	20	6	**650**	December	6	400	
Clarence River	29°25′	1	29	8	**850**	November	7	200	
Wooli River	29°53′	1	20	3	200		3	200	
Bellinger and Kalang Rivers	30°30′	2	29, 14	6	**2400**	November	8	200	
Nambucca River	30°39′	2	48, 43	10	**3200**	November	7	250	
Macleay River	30°52′	2	44, 20	3	50		2	50	
Hastings River	31°25′	3	45, 38, 23	27	**1100**	November	13	**1000**	December
Camden Haven River	31°38′	3	56, 34, 34	29	**650**	November	7	**900**	
Manning River	31°53′	3	53, 52, 41	39	**1700**	October	19	300	May
Wallis Lake	32°13′	3	58, 58, 59	54	**3700**	October	27	**550**	December
Port Stephens	32°42′	10	29, 34, 56, 56, 56, 57, 57, 56, 56, 56	44	**1000**	August	28	**850**	February
Brisbane Water	33°31′	4	55, 52, 37, 56	18	300		24	**1300**	April
Patonga River	33°32′	1	52	4	100		1	50	
Hawkesbury River	33°34′	3	61, 59, 20	14	300		25	350	
Georges River	34°01′	1	57	6	300		4	**500**	March
Shoalhaven and Crookhaven Rivers	34°53′	3	48, 48, 48	30	250		6	100	
Clyde River	35°42′	3	42, 56, 56	9	150		21	250	
Tuross Lake	36°04′	1	53	1	100		-	-	
Wagonga Inlet	36°13′	2	62, 62	27	350		36	**1500**	November
Bermagui River	36°26′	1	23	3	150		-	-	
Wapengo Lake	36°38′	2	57, 56	16	150		1	50	
Nelson Lagoon	36°41′	1	31	1	50		-	-	
Merimbula	36°54′	2	59, 59	15	400		25	**1300**	November
Pambula Lake	36°57′	1	60	11	250		2	100	
Twofold Bay	37°05′	3	48, 48, 48	42	**1400**	March	19	300	
Wonboyn River	37°17′	2	57, 57	18	250		41	**1000**	June

## Supplementary Materials

The following are available online at http://www.mdpi.com/2072-6651/10/11/446/s1, Table S1: Summary of frequency of wild harvest sample collection at Sydney Fish Market during the 2015, 2016 and 2017 wild harvest seasons, Table S2: All active wild harvest beaches along the New South Wales coast during the 2015, 2016 and 2017 wild harvest seasons. The number of wild harvest collection groups operating on each beach during each season is also provided, Figure S1: Images of *Dinophysis* spp. observed in NSW coastal waters under light (A–B and D–F) and calcofluor fluorescence (C,G) microscopy. A–C: *Dinophysis acuminata* D–E: *Dinophysis caudata*, F–G: *Dinophysis tripos*. Images provided by Dr. S. Brett (Microalgal Services), Figure S2: Total weight (kg) of pipis sold each week from north (A) and south (B) coast NSW wild harvest beaches between 2012 and 2017 (data provided by Sydney Fish Market).
