# Supplementary material for: Diarrhetic Shellfish Toxin Monitoring in Commercial Wild Harvest Bivalve Shellfish in New South Wales, Australia
Source: Toxins (Basel). 2018 Oct 30;10(11):446. doi: 10.3390/toxins10110446 (PMC6266617; doi:10.3390/toxins10110446)
Supplement: Supplementary file 1 [file toxins-10-00446-s001.pdf]

Supplementary Materials: Diarrhetic Shellfish Toxin Monitoring in Commercial Wild Harvest Bivalve Shellfish in New South Wales, Australia

Hazel Farrell *, Penelope Ajani, Shauna Murray, Phil Baker, Grant Webster, Steve Brett and Anthony Zammit

Table S1. Summary of frequency of wild harvest sample collection at Sydney Fish Market during the 2015, 2016 and 2017 wild harvest seasons.

Wild Harvest Season	Week(s) of Harvest	Total No. of Samples	Pipi	Cockle	Clam
2015	46	3	3		
	47	4	4		
	48–49	14	12	2	
	49–50	21	19	2	
	51–52, 3	13	13		
2016	25	6	6		
	28	6	5	1	
	33	6	4	2	
	35	6	5	1	
	37	8	5	3	
	39	12	11	1	
	40–41	14	11	3	
	42	6	6		
	43–44	12	10	2	
	44–45	10	8	1	1
	45–46	5	5		
	47–48	17	13	4	
	51–52	14	11	3	
2017	25	4	4		
	29	6	5	1	
	33	6	5	1	
	37	5	3	2	
	38	11	10	1	
	39	10	7	3	
	40	8	7	1	
	40–41	8	8		
	42	5	5		
	43	3	2	1	
	45	11	7	2	2
	46	7	3	4	
	46–47	14	14		
	47–48	16	16		
	49	8	6	2	
	50	15	14	1	
	51	7	4	3	

Table S2. All active wild harvest beaches along the New South Wales coast during the 2015, 2016 and 2017 wild harvest seasons. The number of wild harvest collection groups operating on each beach during each season is also provided.

Wild harvest beach (North–South)	2015	2016	2017
Wooyung Beach	1	1	n/a [1]
South Ballina Beach	2	3	3
Iluka Beach	1	n/a	1
Sandon Beach	n/a	1	n/a
Killick Beach	2	2	2
Goolawah Beach	1	1	1
South Plomer	1	1	1
Lighthouse Beach	2	2	2
Dunbogan Beach	n/a	2	3
Crowdy Head Beach	1	1	1
Tuncurry Beach	1	1	1
Yagon Beach	1	1	1
Big Gibber Beach	1	1	1
Stockton Beach	1	1	1
Seven Mile Beach	n/a	1	1
Bherwerre Beach	1	1	1

[1] n/a = not applicable, wild harvest beach was not opened for collection during annual wild harvest season.

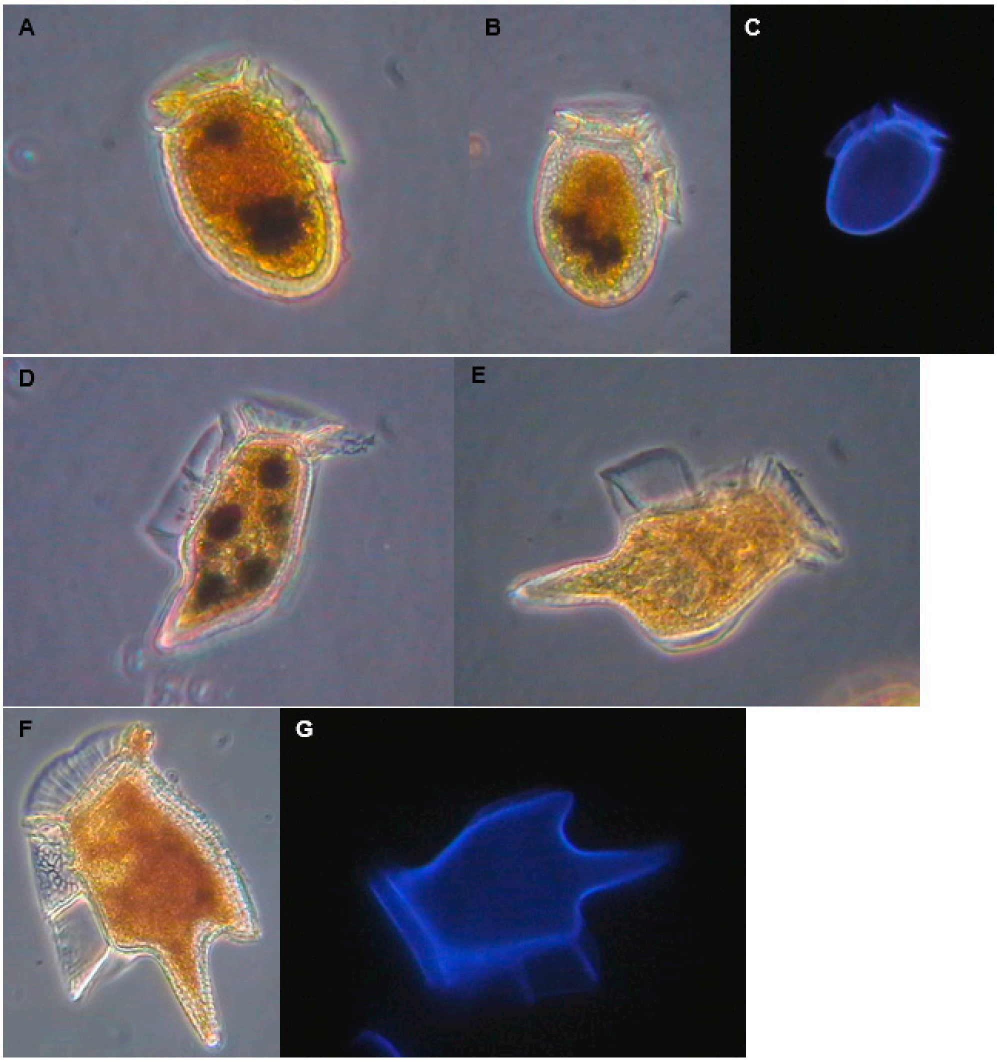

Figure S1. Images of *Dinophysis* spp. observed in NSW coastal waters under light (**A**,**B**,**D**,F) and calcofluor fluorescence (**C**,**G**) microscopy. **A**–**C**: *Dinophysis acuminata* **D**–**E**: *Dinophysis caudata*, **F**–**G**: *Dinophysis tripos*. Images provided by Dr. S. Brett (Microalgal Services).

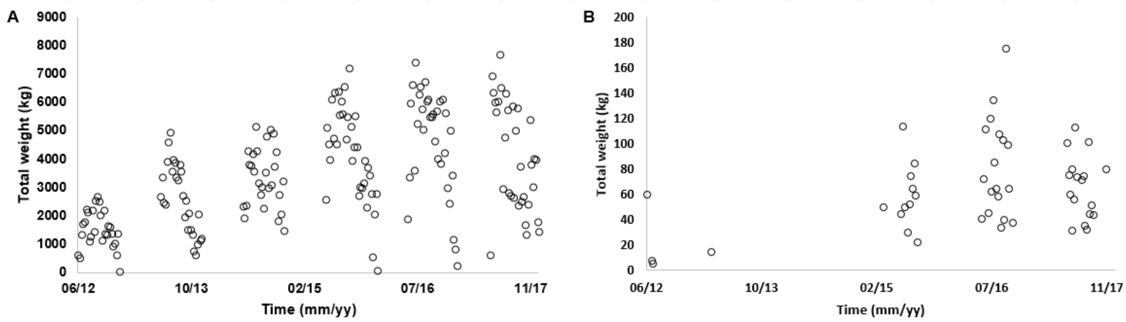

Figure S2. Total weight (kg) of pipis sold each week from north (**A**) and south (**B**) coast NSW wild harvest beaches between 2012 and 2017 (data provided by Sydney Fish Market). Note differing scales of y axes.
